# Cells reprogramming to stem cells inhibit the reprogramming of adjacent cells in the moss *Physcomitrella patens*

**DOI:** 10.1038/s41598-017-01786-1

**Published:** 2017-05-15

**Authors:** Yoshikatsu Sato, Nagisa Sugimoto, Tadayoshi Hirai, Akihiro Imai, Minoru Kubo, Yuji Hiwatashi, Tomoaki Nishiyama, Mitsuyasu Hasebe

**Affiliations:** 1 ERATO, Japan Science and Technology Agency, Okazaki, Japan; 2 Institute of Transformative Bio-Molecules (WPI-ITbM), Nagoya University, Nagoya, Japan; 3 National Institute for Basic Biology, Okazaki, Japan; 4 School of Life Science, Department of Basic Biology, SOKENDAI (The Graduate University for Advanced Studies), Okazaki, Japan; 5 Advanced Science Research Center, Kanazawa University, Kanazawa, Japan; 6 Suntory Global innovation center Limited, Kyoto, Japan; 7 Department of Food Sciences and Biotechnology, Faculty of Life Sciences, Hiroshima Institute of Technology, Hiroshima, Japan; 8 Institute for Research Initiatives, Nara Institute of Science and Technology, Ikoma, Japan; 9 School of Food, Agricultural and Environmental Sciences, Miyagi University, Sendai, Japan

## Abstract

Under certain circumstances differentiated cells can be reprogrammed to form stem cells in land plants, but only a portion of the cells reprograms successfully. A long-distance inhibitory signal from reprogrammed cells to surrounding cells has been reported in some ferns. Here we show the existence of anisotropic inhibitory signal to regulate stem cell formation in the moss *Physcomitrella patens*. When single cells were isolated from a gametophore leaf, over 90% of them were reprogrammed to stem cells with characteristic nuclear expansion. By contrast, when two adjacent cells were isolated, the nuclei of both cells expanded, but successful reprogramming of both cells occurred only in approximately one fifth of the pairs. When three aligned cells were isolated, the reprogramming rate of both edge cells decreased with a living middle cell but did not with a dead middle cell. Furthermore, unequal conversion into stem cells was more prominent in cell pairs aligned parallel to the proximal-distal leaf axis than in those perpendicular to the axis. This study gives an insight into the role of the inhibitory signal in development and evolution as well as the efficient stem cell induction from differentiated cells.

## Introduction

During development, both plants and animals form stem cells, which can self-renew and produce differentiated cells to generate the body^1, 2^. Under certain conditions, differentiated cells can be reprogrammed to regenerate stem cells^3, 4^, which has a significant role in development and reproduction with regard to conferring totipotency and pluripotency. While the regulatory mechanisms of intercellular interactions during reprogramming in a cell population remain largely unknown in seed plants, the physiological aspects of this process have been relatively well studied in fern prothalli, the gametophytic life stage of ferns^5, 6^. Ferns usually have only a single stem cell in the meristem, as opposed to the multiple stem cells found in the meristem of seed plants. In addition, differentiated cells in ferns divide to form a stem cell without advance cell proliferation, which usually occurs before regeneration of stem cells in seed plants^3^. Sections dissected from fern prothallus that lack stem cells typically regenerate a single stem cell^7–9^. Similarly, when the stem cell is ablated in the prothallus, a single stem cell is regenerated^9^. It has been proposed that when the stem cell is lost, all remaining cells are free to become stem cells, but the first to do so inhibits the other cells from becoming stem cells^10^. Two stem cells were formed in approximately 10% of these experiments, cases proposed to represent occurrences of two cells simultaneously reprogramming to stem cells^10^. These findings suggest that stem cells produce an inhibitory signal that is rapidly and efficiently transmitted to surrounding cells^11^. When a single prothallus cell is isolated from adjacent cells by needle ablation, the further the isolated cell is from the original stem cell, the more quickly it reprograms into a stem cell^8^. This observation suggests that the inhibitory factor forms a spatial gradient in the body, such that the concentration near the apical stem cell is higher.

Mosses are one of earliest lineages of land plants^12^ and have the ability to form stem cells from differentiated cells after wounding^13^. Mosses, like ferns, have a single stem cell in the meristem, but they are distinct in that differentiated cells can be directly reprogrammed into stem cells without any cell division^13, 14^. Additionally, in the model moss *Physcomitrella patens* (Physcomitrella), multiple differentiated leaf cells facing a cut are reprogrammed to become putative chloronema apical stem cells within 48 h after dissection, without application of exogenous phytohormones^14^. These differences suggest that the regulatory mechanisms of stem cell formation from differentiated cells in Physcomitrella are distinct from those in ferns.

In this study, we sought to characterize the potential regulatory signal for stem cell formation in Physcomitrella. To this end, we isolated single cells from leaf tissue and showed that the presence of adjacent living cells was not necessary for stem cell formation. We also isolated aligned cell pairs and measured their rate of reprogramming to stem cells to investigate the regulatory mechanism of stem cell formation. The lack of complete reprogramming in both cells of an isolated pair indicated the existence of an inhibitory signal. We further characterized the anisotropic effects of the inhibitory signal and demonstrated that cellular connection is necessary for the inhibitory mechanism, as two cells separated by one dead cell were not inhibited in stem cell reprogramming in either cell. Lastly, we examined the spatial effects of the inhibitory signal by isolating groups of three aligned cells. From these findings we propose that an inhibitory signal for stem cell formation is produced by cells undergoing reprogramming and that this cell fate-dependent signal is anisotropically transmitted.

## Results

### A single isolated leaf cell reprograms to a chloronema apical stem cell

**Figure 1:**
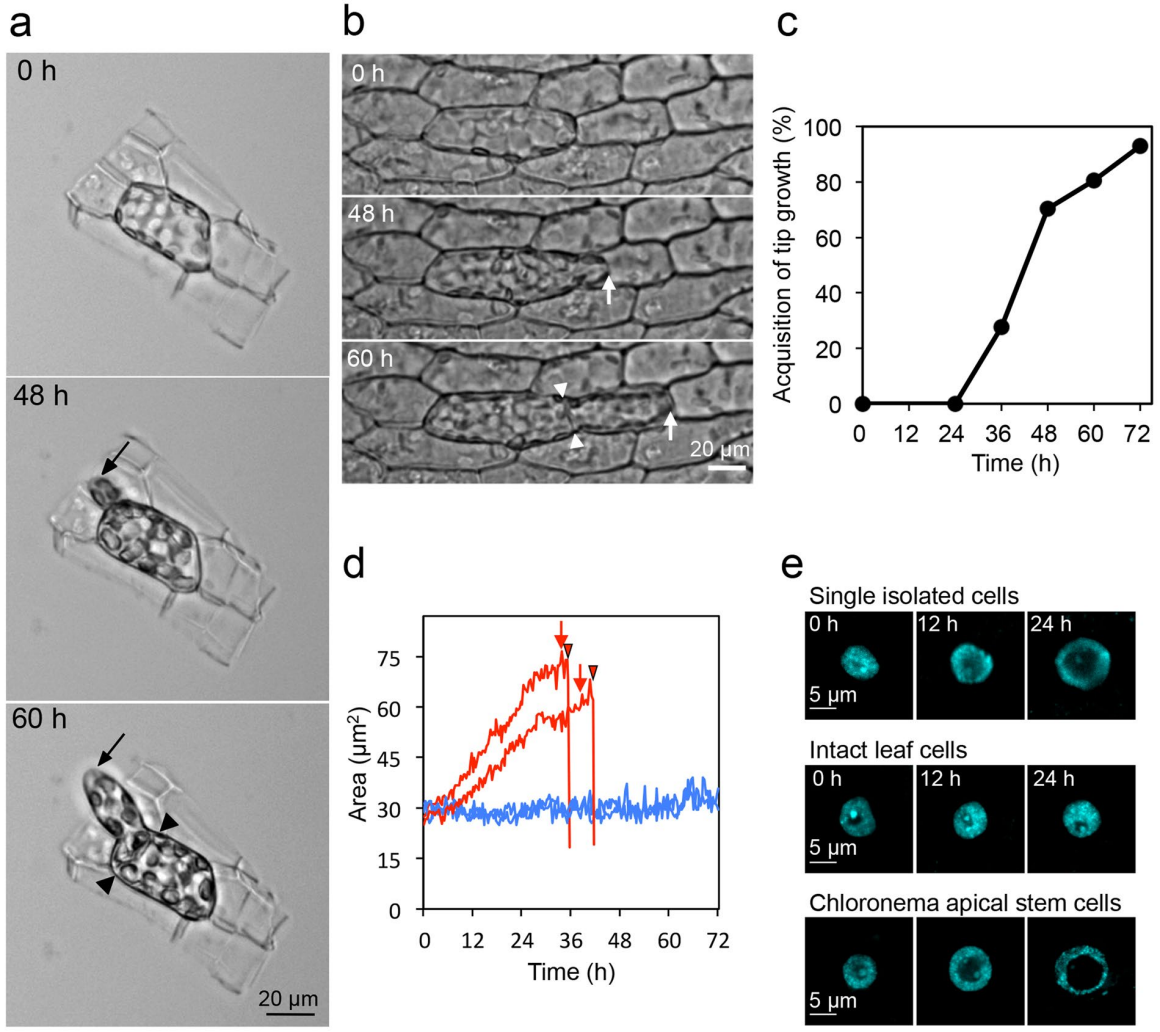
Conversion of an isolated gametophore leaf cell into a putative chloronema apical stem cell. (**a**) Time course images of reprogramming from a single leaf cell isolated with a carbon knife to a putative chloronema apical stem cell. Time after isolation and scales are indicated. (**b**) Time course images of stem cell formation as a single leaf cell isolated by laser ablation converts into a chloronema apical stem cell. Arrows indicate positions of a growing tip and arrowheads indicate a newly formed cell plate. Time after isolation and scales are indicated. (**c**) Percentage of single isolated cells (n = 98) exhibiting tip growth after isolation. (**d**) Changes of nuclear size during stem cell formation, analyzed by tracking HTB2-mRFP signals in single isolated cells (red) and those in intact leaf cells (blue). Arrows and arrowheads indicate time points when tip growth started and when cytokinesis was detected, respectively. (**e**) Fluorescence images of nuclei stained with 4′,6-diamidino-2-phenylindole (DAPI) in single isolated cells, intact leaf cells, and chloronema apical stem cells. Images of chloronema apical stem cells with different size of nuclei are shown. Time after isolation and scales are indicated.

To establish an experimental system in which to examine interactions between cells during stem cell formation in Physcomitrella, we first examined whether an isolated leaf cell can be reprogrammed without adjacent living cells. When we isolated individual single leaf cells by hand using a carbon knife to remove the debris as much as possible, the isolated cell acquired tip-growth and reentered into the cell cycle, indicating that single isolated leaf cells are autonomously reprogrammed to cells that are similar to chloronema apical stem cells (Fig. 1a, Supplementary Video 1). A chloronema apical stem cell continuously divides to regenerate itself and forms a chloronema subapical cell. Therefore, chloronema apical stem cells fulfill the definition of a stem cell: they self-renew and give rise to cells that go on to differentiate. Next, because this method is time-consuming, we isolated single leaf cells by ablating surrounding cells using a laser-microdissection microscope. The resulting single isolated cells mostly displayed tip growth and divided (Fig. 1b), as the single cell isolated by hand and regenerated gametophores within 4 weeks (Supplementary Fig. S1). Overall, 70% of isolated cells (n = 98) protruded at 48 h, and the rate reached 93% at 72 h (Fig. 1c). Tip growth usually proceeded cell divisions (85 out of 91 protruded isolated cells at 72 h). Since nuclear size expansion is associated with reprogramming in metazoans^15, 16^ and we preliminarily observed that the nuclear size of chloronema apical stem cells is larger than that of gametophore leaf cells, we developed a system to measure the size of the nucleus in each isolated cell to examine whether the nuclear size of leaf cells becomes larger before the cell division. A DNA fragment encoding the monomeric red fluorescent protein (mRFP)^17^ was inserted into the Physcomitrella genome just before the stop codon of the *Histone H2B* (*HTB2*) gene. We then observed the nucleus in each isolated cell by tracking HTB2-mRFP signals. We measured the size of isolated cell nuclei through cell division (Fig. 1d) and compared to the size of chloronema apical stem cell nuclei (Supplementary Fig. 2). The nuclear size of chloronema apical stem cells immediately increased after cytokinesis and then gradually increased. Some nuclei prominently swelled just before cytokinesis (Supplementary Fig. 2a). The maximum nuclear size of single isolated cells is significantly associated with the size of chloronema apical stem cell nuclei at the middle time point between cytokinesis but is not with the maximum size (Supplementary Fig. 2b). On the other hand, the nuclear size in intact leaf cells was not enlarged (Fig. 1d, Supplementary Fig. 2b). These results indicate that the nuclear size becomes closer to that of chloronema apical stem cells than that of gametophore leaf cells but the reprogrammed leaf cells with tip growth are not exactly same as chloronema apical stem cells.

In addition, to investigate the size change of nucleoli specifically during stem cell formation, isolated cells were fixed, stained with 4′, 6-diamidino-2-phenylindole (DAPI), and observed with confocal microscopy (Fig. 1e). Both the nucleolus and nucleus expanded during stem cell formation up to 24 h, whose size is similar to that of chloronema apical stem cells (Fig. 1e), although we could not quantitatively analyze the size expansion of nucleoli because quite few isolated cells were stained with DAPI.

**Figure 2:**
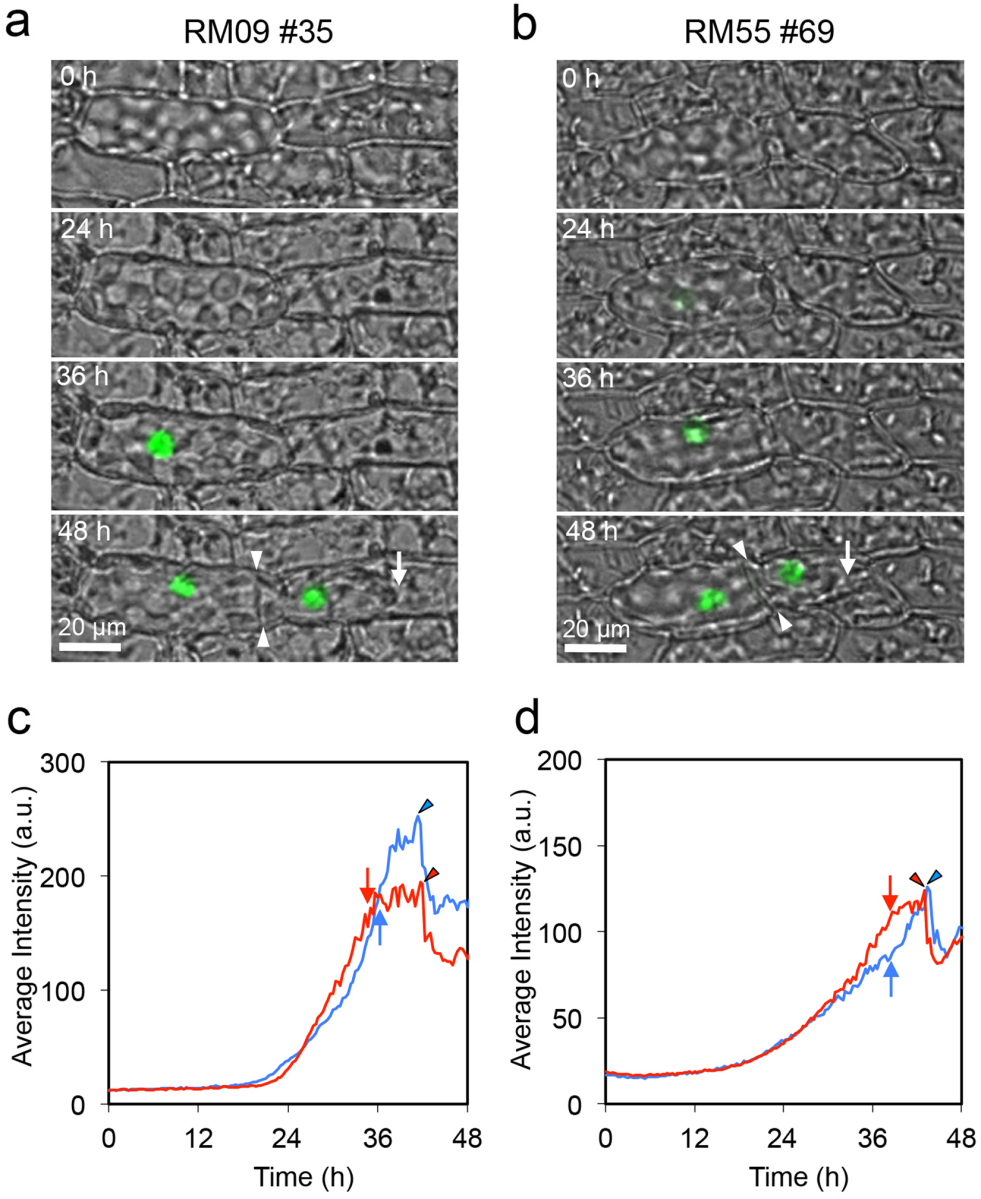
Promoter activities of protonema-specific genes *RM09* and *RM55* in a single isolated leaf cell. (**a**–**d**) Time course images (**a**,**b**) and change of average fluorescence intensity (**c**,**d**) of GFP signals in single isolated leaf cells. Isolated leaf cells of the protonema-specific marker lines (RM09 #35 and RM55 #69) were incubated on BCDAT medium. Twenty-four out of 27 in RM09 #35 and 33 out of 41 isolated cells in RM55 #69 showed tip growth until 72 h. *RM09* and *RM55* genes are specifically expressed in protonemata and not in leaf cells^14^. Images were taken at 20-min intervals for 48 h. Fluorescence images of GFP (green) are overlaid with bright field images (**a**,**b**). Time after isolation and scales are indicated. Arrows and arrowheads in (**a**) and (**b**) indicate positions of tip growth and newly formed cell plates, respectively. Those in (**c**) and (**d**) indicate time points when tip growth started and when cytokinesis was detected, respectively. Measurements from two independent cells are shown in red and blue (**c**,**d**).

To examine when an isolated leaf cell acquires chloronema cell fate, we isolated single leaf cells from transgenic lines harboring *green fluorescent protein* (*GFP*) expressed from the promoter of either *RM09* or *RM55*, each of which is specifically expressed in protonemata but not in leaf cells^14^. In both promoter reporter lines, GFP signal became detectable approximately 24 h after isolation, before tip growth and cell division (Fig. 2, Supplementary Videos 2 and 3). These results indicate that a single isolated leaf cell acquires some characteristics of chloronema cells before tip growth and cell division, as is found in dissected leaf cells during reprogramming^14^.

### A differentiated leaf cell converting to a stem cell anisotropically inhibits an adjacent cell from reprogramming to a stem cell

**Figure 3:**
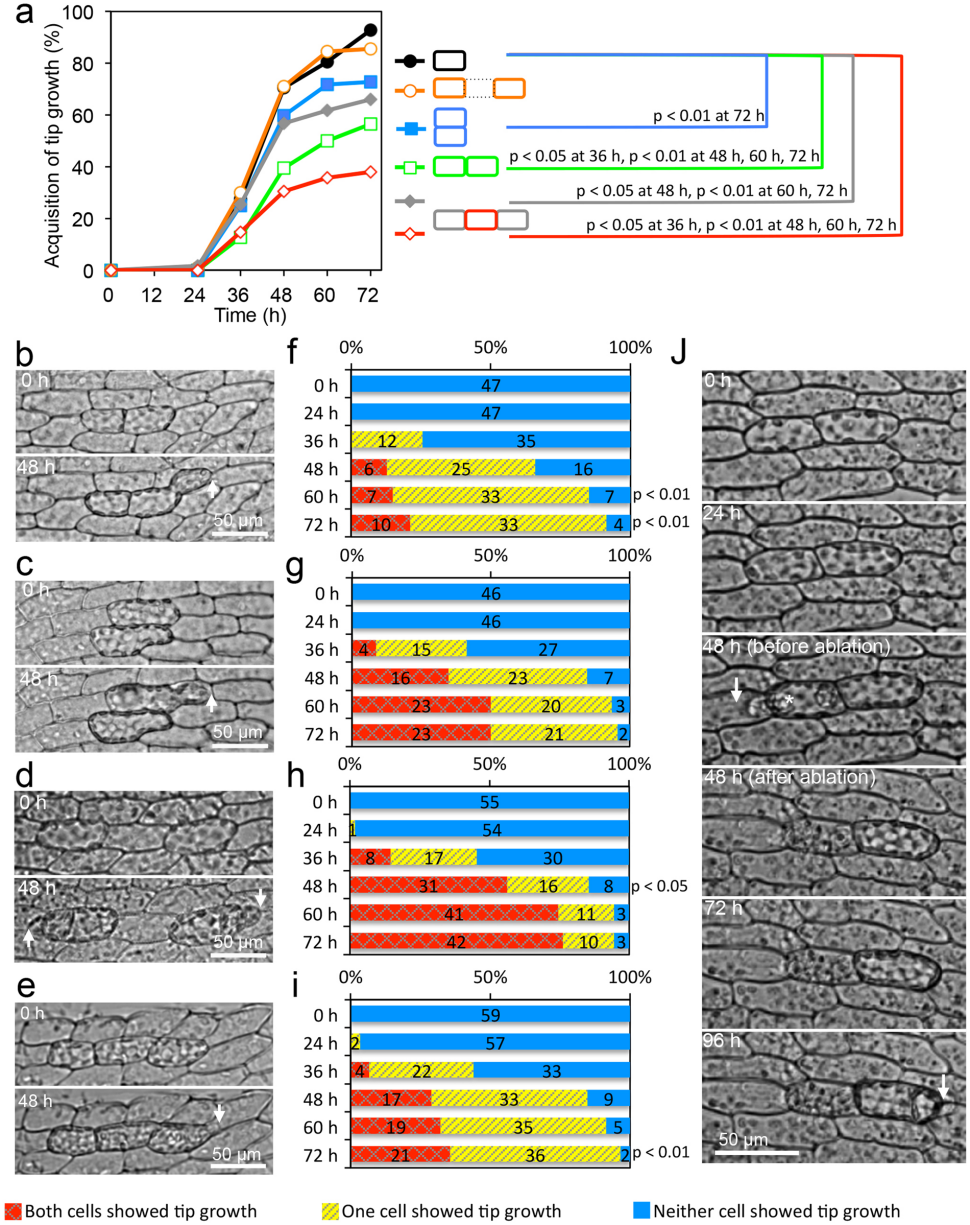
Intercellular communication during stem cell formation. (**a**) Percentages of the number of cells with tip growth in the total number of examined cells in each type of isolated cells. Single isolated cells (black solid circle, n = 98 single isolated cells, identical to Fig. 1c), two cells separated by a dead cell (orange open circle, n = 110 edge cells in 55 three aligned cells), two cells aligned parallel (green open square, n = 94 edge cells in 47 paired cells) or perpendicular (blue solid square, n = 92 edge cells in 46 paired cells) to a proximal-distal leaf axis, middle cells in aligned three cells (red open rhombus, n = 95 middle cells in 95 three aligned cells), and both edge cells of three aligned cells without tip growth in middle cells (gray solid rhombus, n = 118 edge cells in 59 three aligned cells) were examined. Statistical significance of difference from single isolated cells was examined by Fisher’s test. (**b**–**i**) Images (**b**–**e**) and cell fates (**f**–**i**) in isolated pairs of cells aligned parallel (**b**,**f**) or perpendicular (**c**,**g**) to the leaf proximal-distal axis, isolated pairs of cells separated by a dead cell (**d**,**h**), and isolated sets of three cells aligned perpendicular to the axis and without tip-growth in a middle cell (**e**,**i**). Arrows indicate the position of a growing tip. Time after isolation and scales are indicated. In (**f**–**i**), the percentages of cell pairs with no tip growth in both edge cells (blue bar), tip growth in one cell (yellow hatched bar), and tip growth in both cells (red crosshatched bar) are shown. The numbers of cells are indicated within the bars. Significance was determined by exact tests of the Hardy-Weinberg equilibrium. (**j**) Effect of ablation of a reprogrammed stem cell on the adjacent cell in isolated pairs of aligned cells. Two cells parallel to the leaf axis were isolated and incubated on BCDAT medium. When one of the two cells protruded at 48 h after isolation, the protruded cell (asterisk) was ablated. The arrows indicate the position of a growing tip. Time after isolation and scales are indicated.

To investigate the presence of inhibitory signals for stem cell reprogramming between two adjacent cells, we isolated pairs of adjacent cells and measured the rate of successful reprogramming (Fig. 3a). We isolated two adjacent cells aligned either parallel or perpendicular to the proximal-distal leaf axis by laser microdissection (Fig. 3b,c). At 72 h after isolation, 93% of single isolated leaf cells (91 out of 98 cells) exhibited tip growth, as an indication of stem cell fate, whereas only 56% (53 cells out of 94 cells in 47 parallel pairs) and 73% (67 cells out of 92 cells in 46 perpendicular pairs) of paired cells displayed tip growth (Fig. 3a). Overall, the reprogramming percentages of cells isolated as pairs were significantly lower (p = 3.9E-09 and p = 3.6E-04) than that for single isolated cells (Fig. 3a). To investigate positional tendency of reprogramming, we counted the number of parallel and perpendicular pairs, in which only one cell is reprogrammed. Of 33 parallel pairs, 20 and 13 cells in the proximal and distal sides were protruded, respectively, which is not significantly different from 1: 1 ratio (p = 0.30, binomial test). Of 21 perpendicular pairs, 13 and 8 cells in the leaf-vein and leaf-edge sides were protruded, which is not significant as difference (p = 0.38, binomial test).

However, when the two cells isolated parallel to the proximal-distal axis were separated by a dead cell (Fig. 3d), the reprogramming rate was not significantly different from that in single isolated cells (85% [94 in 110 cells of 55 separated pairs], p = 0.12) (Fig. 3a). These results indicate that stem cell formation is negatively regulated by intercellular communication between adjacent cells. As the cells are not well aligned in the direction perpendicular to the leaf axis and the bordering regions vary between cells in that direction, experiments with three aligned cells were conducted only with cells aligned parallel to the leaf axis.

Next, we investigated whether an inhibitory signal originates from a reprogramming stem cell according to the hypothesis that a stem cell prevents the other cells from becoming stem cells. A null hypothesis is that there is no stem cell-specific signal and the reprogramming inhibition is independent from the cell fate. If there were no stem cell-specific signal, the fate of each cell would be independent from the fate of the adjacent cell. In such a case, if the probability of a cell to be reprogrammed as a stem cell is *p*, then the probability of having both cells reprogrammed, one of the cells reprogrammed, and neither cell reprogrammed would be *p* ^2^, 2*p*(1-*p*), and (1-*p*)^2^, respectively. These terms are the same as those for allele and genotype frequencies in the Hardy-Weinberg equilibrium^18, 19^, and deviation from this expectation can be tested with the same method. The reprogramming frequencies in two connected cells aligned parallel to the leaf proximal-distal axis showed significant deviation (p = 0.007 at 72 h) from the expectation under the assumption of independent reprogramming, with an excess (70%, 33 out of 47 pairs) of cases where only one of two cells was reprogrammed (Fig. 3f). These results indicate that a cell fate-dependent signal does exist, when two connected cells are aligned parallel to the leaf proximal-distal axis. However, when two cells were aligned perpendicular to the leaf axis (Fig. 3c), we could not detect significant cell fate-specific inhibition (Fig. 3g, p = 0.46 at 72 h), even though stem cell formation is negatively regulated by intercellular communication between adjacent cells (Fig. 3a). These results suggest that cell fate-dependent inhibitory signal is transmitted parallel to the leaf axis more readily than perpendicular to the leaf axis. In addition, the percentage of cell pairs with tip growth in only one cell was higher in pairs of adjacent cells (70%, 33 out of 47 parallel pairs; Fig. 3f) than that in pairs in which the two cells were separated by a dead cell (18%, 10 out of 55 pairs; Fig. 3h). Thus, cellular connection is necessary for the cell fate-dependent inhibition. To confirm whether a cell fated to be a stem cell suppresses the other cell to be a stem cell, we ablated a reprogrammed stem cell already exhibiting tip growth among two aligned cells. The non-ablated remaining cell protruded within 48 h after ablation of the adjacent cell (8 out of 9 cells), further supporting the hypothesis that the inhibition depends on the adjacent reprogramming cell (Fig. 3j, Supplementary Video 4). To investigate the effects of phytohormones in the cell fate-dependent inhibition, we exogenously supplied 1 µM of naphthaleneacetic acid (NAA) and benzylaminopurine (BAP), and strigolactone analog GR24 to isolated pairs of cells aligned parallel to the leaf axis (Supplementary Fig. 3). The cell fate-dependent inhibition was detected with statistical confidence in all the phytohormones. However, the number of reprogrammed cells decreased with exogenous strigolactone (Supplementary Fig. 3a and d), suggesting that strigolactone inhibits the reprogramming process but is not involved in the cell fate-dependent inhibition.

### Inhibitory signal is transmitted beyond a single cell range

To determine the range of the inhibitory signal for stem cell reprogramming, we isolated three adjacent cells parallel to the leaf axis (Fig. 3e). Of the three adjacent cells, stem cell formation of the middle cell was strongly suppressed, with only 38% (36 in 95 triplets) of middle cells successfully reprogramming in isolated triplets at 72 h after isolation (Fig. 3a). Among the 59 triplets without tip growth in the middle cells, either one of the two edge cells acquired tip growth in 61% (36 in 59 triplets) of the isolated triplets, while both edge cells protruded in 36% (21 in 59 triplets) of the triplets (Fig. 3i). These results suggest that tip growth, and therefore stem cell reprogramming, in the edge cells was subject to the fate of the other (p = 0.008 at 72 h). Thus, the range of the stem cell fate-specific inhibitory signal appears to expand beyond at least one living cell. It was not possible to examine how far the inhibitory signal expands, since more than three cells are not well aligned.

### Inhibition acts after nuclear expansion and before DNA synthesis

**Figure 4:**
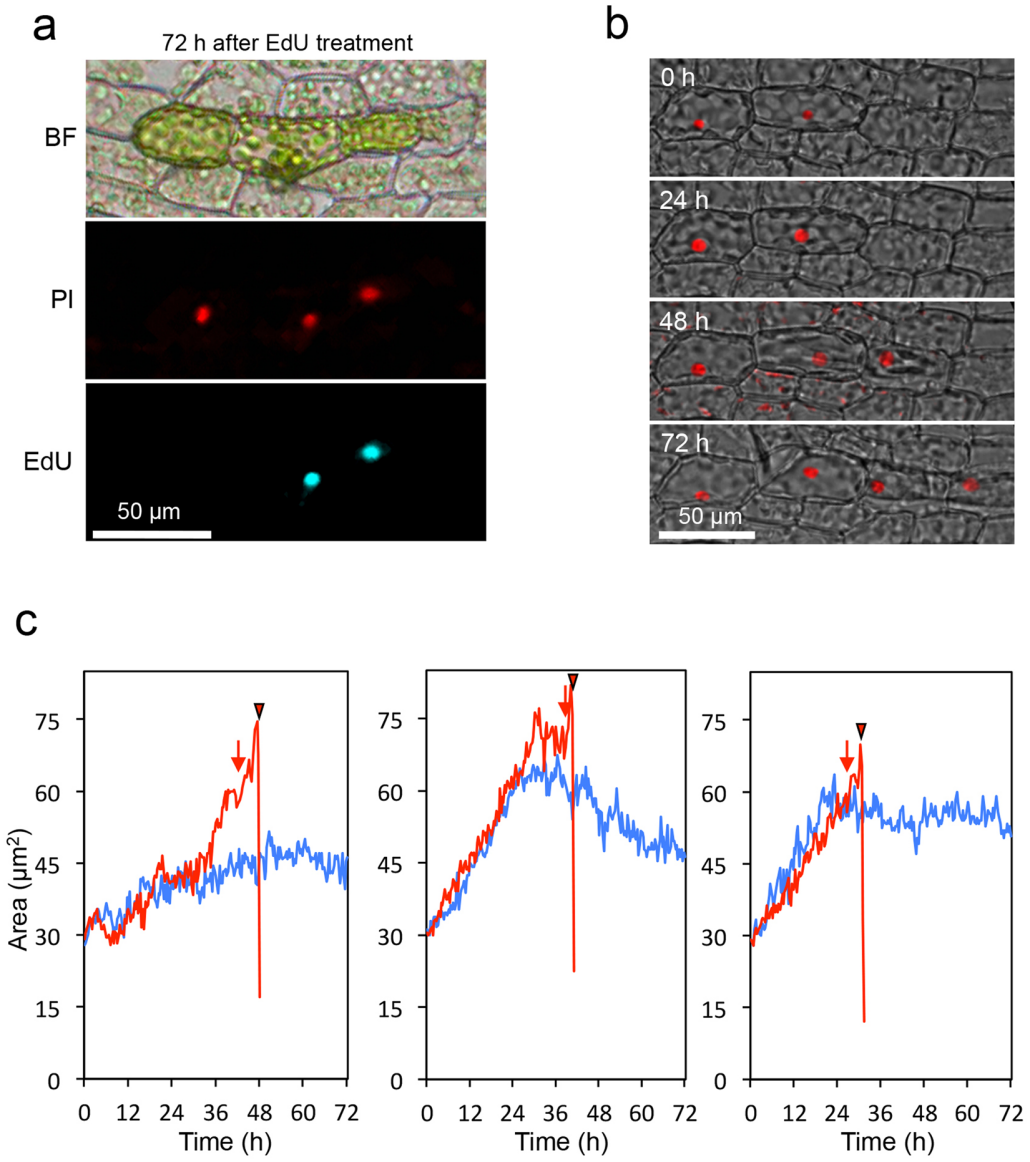
Changes of nuclei in isolated two adjacent cells. (**a**) Bright field (BF), propidium iodide (PI) fluorescence (red), and 5-ethynyl-2′-deoxyuridine (EdU) fluorescence (cyan) images of two adjacent cells incubated with 30 µM EdU for 72 h after isolation. (**b**) Sequential fluorescence images of the HTB2-mRFP signal were overlaid with bright-field images in isolated two adjacent cells aligned parallel to the leaf axis. Time after isolation is indicated. (**c**) Nuclear size analyzed by tracking the mRFP signal in isolated pairs of adjacent cells aligned parallel to the leaf axis. Nuclear sizes of cells with (red and magenta) or without (blue and cyan) tip growth and cell division are indicated. Arrows and arrowheads designate the time of commencement of tip growth and cell division, respectively.

To better understand the mechanism of reprogramming inhibition, we next set out to determine the point at which the inhibitory signal acts to prevent stem cell formation. The cell cycle in mature leaf cells in Physcomitrella is arrested at the late S phase, with DNA synthesis resuming before cell division^14^. When we applied 5-ethynyl-2′-deoxyuridine (EdU)^20^ to the cells to visualize DNA synthesis, the signal was specifically detected in cells with tip growth and cell division in all cases out of 21 cell pairs (Fig. 4a). These results indicate that the inhibition of stem cell formation occurs at least before cell cycle re-entry into the S phase.

Since the nucleus expanded during stem cell formation in a single isolated cell (Fig. 1d), we examined whether isolated pairs underwent nuclear expansion using the HTB2-mRFP lines. We found that the nucleus of both cells in a pair exhibited nuclear expansion and the maximum nuclear size in cells with tip growth was larger than that in cells without tip growth on average (Supplementary Fig. 2b). Following this expansion, the nuclear size reduced over time in cells that did not become stem cells while the nucleus in cells that acquired stem cell fate proceeds to divide (Fig. 4b,c, Supplementary Fig. 2, Supplementary Video 5). Quantitatively, nuclear size at 72 h in cells without showing tip growth (49.4 ± 9.1 µm^2^) is significantly smaller than that at time of nuclear division in cells with tip growth (54.4 ± 10.3 µm^2^) by paired t-test (p = 0.003, n = 20). Together, these results indicate that cells that do not ultimately acquire stem cell fate are also partially reprogrammed and that the inhibitory signal acts after nuclear expansion and before cell cycle reentry into the late S phase.

**Figure 5:**
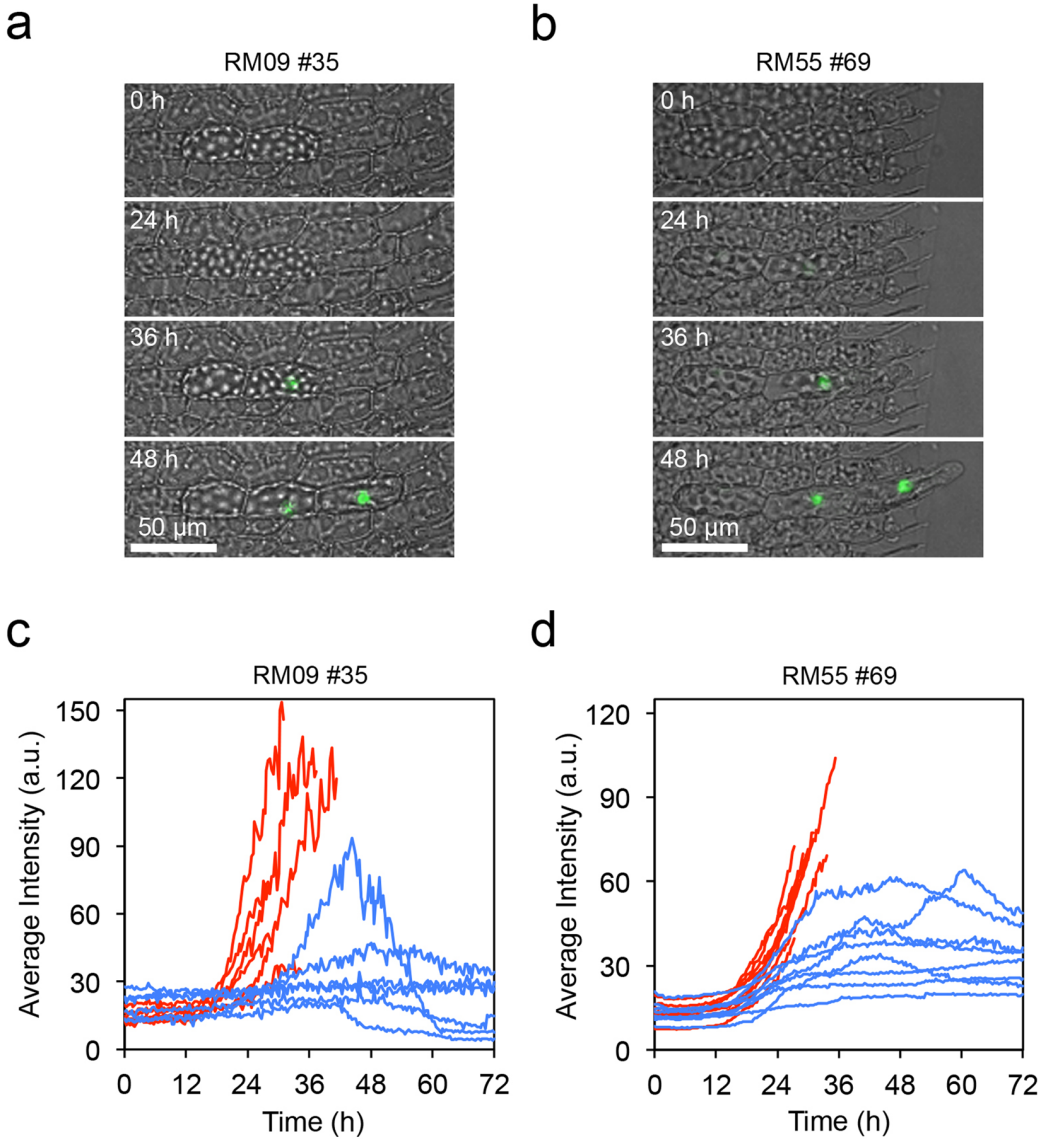
Promoter activities of protonema-specific genes *RM09* and *RM55* in isolated two adjacent cells. (**a**–**d**) Time course images (**a,b**) and changes of average fluorescence intensity (**c**,**d**) of GFP signals in isolated adjacent cells in RM09 #35 (**a,c**) and RM55 #69 (**b**,**d**) lines, respectively. Thirty-two out of 63 and 9 out of 26 pairs showed tip growth in only one cell. GFP (green) are overlaid with bright field images (**a**,**b**). Time after isolation and scales are indicated. Measurements of average intensity from independent cells (n = 6 in (**d**), n = 8 in (**e**)) are shown until the time of tip growth acquisition (**c**,**d**). Cells with and without tip growth were indicated in red and blue, respectively (**c**,**d**).

We also examined the protonema marker expression in cell pairs aligned parallel to the leaf axis using RM09 and RM55 lines. GFP signal was accumulated only in cells with tip growth in some cell pairs (Fig. 5a,b, Supplementary Videos S6 and 7), while other pairs acquired GFP signal in both cells with and without tip growth (Fig. 5c,d). In the latter pairs, GFP signal diminished in cells not to acquire tip growth (Fig. 5c,d). Therefore, the reprogrammed cells inhibit the reprogramming of neighboring cells even after the expression of protonema marker genes. In edge cells of three aligned cells with living middle cells, GFP signal accumulated in cells with tip growth. In cells without tip growth, GFP signal did not accumulate or diminished after accumulation (Supplementary Fig. 4a,b). On the other hand, GFP signal accumulated in both cells to become stem cells in cell pairs separated by dead cells (Supplementary Fig. 4c,d). It should be noted that the reprogramming frequency of two connected cells in RM55 did not show significant deviation from the expectation under the assumption of independent reprogramming (the number of cell pairs with tip growth in both cells: 17, that in one cell: 9, and that in no cells: 0 at 72 h). Also, those of edge cells in living triplet cells in RM09 did not (the number of cell pairs with tip growth in both cells: 26, that in one cell: 13, and that in no cells: 2 at 72 h). In addition, 58% (56 out of 97 triplets) of the middle cells showed tip growth at 72 h in living triplet cells of RM09, which is higher than 38% (n = 95) in wild type (p = 0.006). Since cellular damage enhances the reprogramming, absorbed photons to fluorescence proteins might cause cellular damage.

## Discussion

In this study, we established a single-cell isolation system in Physcomitrella using laser microdissection and found that single isolated cells autonomously initiated tip growth and cell division (Fig. 1). The resulting cells were similar to chloronema apical stem cells in terms of their morphology (Supplementary Fig. 1) and expression of protonema-specific genes (Fig. 2), although the maximum nuclear size is approximately 15% smaller than that of chloronema apical stem cells (Supplementary Fig. 2b). This process is similar to the stem cell formation that takes place in dissected Physcomitrella leaves, in which cells facing the cut edge undergo reprogramming into putative chloronema apical stem cells^14^. This single-cell system will be useful in studying the reprogramming of differentiated cells into stem cells in the absence of potentially confounding effects of surrounding cells. In the fern *Pteris vittata*, a single isolated prothallus cell is converted into a rhizoid cell that exhibits tip growth but does not self-renew^8^. Only subsequently does the converted rhizoid cell divide to form an apical stem cell with the ability to self-renew and produce differentiated cells. Our results support the idea that ferns and mosses use different mechanisms to regenerate stem cells from differentiated cells, although both can regenerate stem cells from a single differentiated cell.

In ferns, the fact that only a single apical stem cell is regenerated on a dissected gametophyte segment is interpreted to mean that the first regenerated stem cell releases an inhibitory signal repressing stem cell formation in the entire segment^10^. Physcomitrella, by contrast, is capable of forming multiple stem cells in a single gametophyte leaf tissue section^14^. To explore the process underlying this higher proportion of reprogrammed cells, we examined the inhibitory mechanism of stem cell formation in Physcomitrella by isolating two adjacent cells. We found that the rate of stem cell formation in either of the two aligned cells was lower than that for single isolated cells (Fig. 3a). Moreover, the separation of two cells by a dead cell resulted in recovery of the stem cell reprogramming rate (Fig. 3a), indicating that an inhibitory signal is produced in one or both of the isolated cells and that the signal transmission needs a living adjacent cell. The frequency of one cell in an isolated pair displaying tip growth was higher than would be predicted for independent reprogramming (Fig. 3f). This indicates the presence of cell fate-dependent inhibition between the two cells, i.e., a reprogramming cell produces an inhibitory signal received by the other cell. Furthermore, the inhibitory signal is not completely exclusive (Fig. 3f), which is different from the inhibitory signal in ferns^10^. When one cell displayed tip growth characteristic of reprogramming while the second cell did not, ablation of the protruded cell induced protrusion in the second cell, which is interpreted to indicate that the second cell was freed from the inhibition signal by ablation of stem cell (Fig. 3j). This supports the hypothesis that an inhibitory signal is transported from the reprogramming cell to the other cell. However, cell fate-dependent inhibition of the edge cells was detected in two-cell pairs aligned to the leaf proximal-distal axis but not in perpendicular pairs. Together, these results indicate that the inhibitory signals from cells that acquired stem cell fate are anisotropically transmitted. Exogenous supplementation of auxin, cytokinin, and strigolactone did not affect the cell fate-dependent inhibition in this study, although more detailed studies will be necessary in future to rule out the possibility of the involvement of these phytohormones. On the other hand, anisotropically transported proteins may function as the inhibitory signal, as the photoconverted protein Dendra2 was found to be anisotropically transported in Physcomitrella leave^21–23^.

Typically, studies on reprogramming focus specifically on the differentiated cells undergoing reprogramming to stem cells^9^ while the cellular state of the cells that remain unchanged are not closely examined. In this study, nuclear expansion was detected in both cells of pairs of isolated Physcomitrella leaf cells (Fig. 4b,c, Supplementary Fig. 2) but DNA synthesis occurred only in the cell destined to become a stem cell (Fig. 4a). Furthermore, we found that the expanded nucleus of non-stem cell began to shrink back toward its original size when the ultimate stem cell showed tip growth (Fig. 4c). Together these findings imply that (1) established stem cells not only inhibit stem cell formation in adjacent cells but also bring back adjacent cells to a differentiated state, or (2) cells that fail to become stem cells autonomously return to a differentiated state. This hypothesis is concordant with the expression changes of protonema marker genes (Fig. 5).

Although many details of the molecular mechanism by which stem cells inhibit reprogramming of neighboring cells remain unknown, the inhibitory signals are likely involved in normal development as well as regeneration. Identification of the inhibitory molecule in the future should provide insight into the mechanism of inhibition. The identity and mechanism of action of the inhibitory signal is also interesting in terms of the evolution of multicellularity. Multicellular organisms evolved to have different cell types, some of which do not contribute to descendants. This ability to generate stem and non-stem cells is fundamental to the evolution of multicellularity. Having established a single-cell system for studying stem cell reprogramming, we hope to use this system to improve our understanding of the molecular nature of this developmental process in the near future.

## Methods

### Plant materials and growth conditions

The moss *Physcomitrella patens* Gransden 2004 strain^24^ was used as a wild type and cultured for 4 to 5 weeks on BCDAT medium under continuous white light at 25 °C^25^. The third to fifth gametophore leaves from the apex were used for reprogramming analysis. For observation of chloronema apical stem cells, protonemata were cultured on BCDAT medium for 3 to 5 days.

### Plasmid construction for the HTB2-mRFP plasmid

To generating knock-in lines with reporter fusions of the *histone H2B* (*HTB2*) gene, the monomeric red fluorescent protein gene *mRFP* ^17^ was inserted via gene targeting to fuse in frame with *HTB2* through a connection with the flexible linker peptide GAGAGAGAGA^26^. An oligonucleotide including sequences encoding the flexible linker peptide and multiple cloning sites was synthesized with primers 5′-CGGCCGCGGCCGCGGCCGCAGGGGCCGGTGCAGGGGCTGGAGCAGGGGCAGCTGCTGCCT-3′, 5′-CTGGACCCAACGCATGCCTGGGCACCACTAGTTCACTATTAGGCAGCAGCTGCCCCTGCT-3′, 5′-GGGCAATTAATTAAGTTTAAACATTTAAATGGGGCCGTCCCGGCCGCGGCCGCGGCCGCA-3′, 5′-GGCCTTAATTAAGTTTAAACCATTTAAATCCACCGGTCCGCTGGACCCAACGCATGCCTG-3′, 5′-CCGAGCTCGCCCGGGCAATTAATTAAGTTTAA-3′ and 5′-GGGTACCGCCCGGGCCTTAATTAAGTTTAAAC-3′. The DNA fragment was inserted to the pBluescript II SK plus vector (Stratagene) between the *Kpn*I and *Sac*I sites. Full-length *mRFP* amplified with primers 5′-TTGCCGCTGCGGCTGCCTCCTCCGAGGACGTC-3′ and 5′-CCTAGCTAGCGGCGCCGGTGGAGTGGC-3′ was inserted between *Bst*API and *Spe*I sites to construct a pFlex-mRFP plasmid. To construct a HTB2-mRFP plasmid, the 3′ genomic DNA fragment of *HTB2* was amplified with primers 5′-AGACCTCCAGCAGTTGTGCCGATCC-3′ and 5′-ATTCCCATGTGTGAATTGTCGCTGA-3′ and inserted into pFlex-mRFP at the *Xcm*I site, resulting in pFlex-mRFP-HTB2-3′. The 5′ genomic fragment of *HTB2* was amplified with primers 5′-AGGCCGTCCCGGCCACACAAACAACTCGAAATCCAAAAC-3′ and 5′-AGGCCCCTGCGGCCCCAGCGCTAGTAAACTTAGTGACGGCT-3′ and inserted into pFlex-mRFP-HTB2-3′ at the *Sfi*I site to construct pHTB2-mRFP.

The plasmid sequence is deposited in DDBJ (AB969737).

### Gene targeting without selection marker insertion

Transformation to generate the PpHTB2-mRFP lines was performed without selection marker insertion (Supplementary Fig. 5a). HTB2-mRFP plasmid (10 µg) was linearized with *Pme*I and mixed with an equivalent amount of *Sal*I-digested pTN182 (Accession number: AB267706) containing a *neomycin phosphotransferase II* (*nptII*) expression cassette^25^, and used for transformation. Transformed protoplasts were selected on BCD medium with 20 µg/mL G418 medium for 2 weeks. Regenerated protonemata with mRFP signal were visually selected and transplanted on BCDAT medium without G418 and cultured for a week. After one-week cultivation, all protonemata became sensitive to G418. To eliminate polyploid lines, flow cytometry analysis was performed as described previously^27^. The candidates were further analyzed by DNA gel-blot analysis^28^ to exclude transformants with tandem integrated transgenes, and three independent lines with a single insertion of *mRFP* in the *HTB2* locus were obtained (Supplementary Fig. 5b). Development in these transgenic lines was not distinguished from that in wild type, and fluorescence with emission characteristics of mRFP was detected in the nuclei of gametophore leaf cells, chloronema cells, and caulonema cells (Supplementary Fig. 5c–e). The mRFP-labeled nuclei were excited with a green excitation band-pass filter unit (U-MRFPHQ; Olympus). The fluorescence images were acquired using an inverted microscope (IX-81; Olympus) with a 20X objective lens (UPlanSApo20X, NA 0.75; Olympus) through a cooled CCD camera (ORCA-AG, Hamamatsu Photonics K. K.). A confocal microscope (LSM 780; Zeiss) with a 20X objective lens (Plan Apochromat, NA0.80; Zeiss) was also used for observation of HTB2-mRFP expression in gametophore leaf cells. The fluorescence of mRFP and chlorophyll were excited with 560 nm excitation laser and collected with 570–658 nm and 672–701 nm emission spectra, respectively. The rate of stem cell formation was not distinguishable from that in wild type (Supplementary Fig. 5f).

### Isolation of leaf cells with laser dissection microscope

Leaves of 4- to 5-week-old gametophores were excised and divided into 6 segments with a carbon knife. All cells not targeted for reprogramming were ablated by irradiating just one chloroplast with a solid-state ultraviolet laser (355 nm) through a 20X objective lens (LD Plan-NEOFLUAR, NA 0.40; Zeiss) at a laser focus diameter of less than 1 µm using the laser pressure catapulting function of the PALM microdissection system (Zeiss). The analysis was performed in targeted cells which survived for 72 h after isolation. A chloroplast to irradiate was chosen at a position distantly located from the targeted cell to minimize the irradiation effect.

### Nuclear staining

Gametophore leaves and protonemata were fixed in 3.7% (w/v) formaldehyde for 30 min in 50 mM sodium phosphate buffer (pH 7.0). Samples were immersed in cold absolute methanol for 10 min and treated with 1% Triton X-100 for 30 min. Cells were subsequently stained with 0.1 g/mL 4′, 6-diamidino-2-phenylindole (DAPI) for 30 min. Confocal microscopes (LSM 780; Zeiss) were used with a 63X oil immersion lens (Plan-Apochromat, NA 1.40; Zeiss) to observe the fluorescence. DAPI-stained nuclei were excited with a 405-nm excitation laser, and the fluorescence between 420 and 480 nm was detected. For detection of DNA synthesis, excised leaves were incubated in liquid BCDAT medium containing 30 µM 5-ethynyl-2′-deoxyuridine (EdU; Invitrogen) for 72 h and fixed with 3.7% (w/v) formaldehyde for 30 min. Leaves were rinsed with 1% BSA in PBS (137 mM NaCl, 2.7 mM KCl, 1.5 mM KH_2_PO_4_, 8.0 mM NaHPO_4_) and treated with 1% Triton X100 for 30 min. Cells were incubated with 100 µg/ml RNaseA for 30 min at 37 °C and immersed with Click-iT reaction solution (Invitrogen) for 30 min. After rinsing with 1% BSA in PBS, stained with 1 µg/mL propidium iodide (PI) containing 10 µg/ml RNaseA. Observations were made using an Axiovert 200 M microscope (Zeiss) with a 20X objective lens (LD Plan-Neofluar, NA 0.40; Zeiss) equipped with a CCD camera (AxioCam MRc; Zeiss). The excitation filter BP450–490 and the emission filter BP515–565 were used for EdU signal detection and the excitation filter BP510–560 and the emission filter LP590 were used for PI observation.

### Timelapse analysis

To elucidate the process of stem cell formation from single cells, we established a fluorescence multi-position timelapse system. Isolated leaf cells were placed on a well of a 96-well plate with BCDAT liquid medium. Alternately, isolated cells were placed on a 35-mm glass-bottom dish with 2% methylcellulose and covered with BCDAT agar medium separated with a sheet of cellophane. Timelapse analysis was performed in BCDAT liquid medium at 10 or 20 min intervals through a cooled CCD camera (ORCA-AG, Hamamatsu Photonics K. K.) or a EMCCD camera (Evolve 512 and Cascade II 512, Photometrics) under an inverted microscope (IX71 and 81, Olympus) equipped with a 20X objective lens (UplanSApo, NA 0.75 or LCUPlanFLN, NA 0.45; Olympus). Since light is necessary for the reprogramming to stem cells (Chopra and Kumra, 1988), a light-emitting diode (CA-DSW7: Keyence Corp. or TH-100X100SW and FPR-100SW2, CCS Inc.) was used as the source of white light (approximately 20 µmolm^−2^s^−1^). For fluorescence timelapse imaging analysis, illumination was automatically switched from “on” state to “off” state at fluorescence image acquisition. A blue excitation band-pass filter unit (U-MNIBA3, Olympus) and a green excitation band-pass filter unit (U-MRFPHQ, Olympus or TRITC-A-Basic, Semrock) were used for GFP and mRFP observation, respectively. We acquired 9–15 z-planes with 1.5–2.0 µm interval in a HTB2-mRFP line, and one or 5 z-planes with 4 µm interval in RM lines to choose an appropriate focal plane. The images were reconstructed to create a movie with image analysis software of MetaMorph (Molecular Devices) and ImageJ (http://imagej.nih.gov/ij/). For correction of sample drifting movement during timelapse observation, frames were aligned using the StackReg plugin in ImageJ^29^. The average intensity of cells and nuclear size was also measured by MetaMorph and ImageJ. The average intensity of the signal at each time point was calculated as the intensity of the GFP signal subtracted by the background intensity that was determined as the mean GFP channel intensity of a circular area where no cell was present. The nuclear size was calculated at each time point using the default method of ImageJ based on IsoData algorithm. The merged images and videos between bright-field and fluorescenct images were prepared with ImageJ by taking difference of GFP and RFP channel data and overlaying with bright field images.

### Image processing of color bright-field image

Color bright-filed images from single cell to regenerating gametophore were observed under an inverted microscope (Axiovert 200 M; Zeiss) and a stereomicroscope (SZX16; Olympus) equipped with CCD cameras (AxioCam MRc; Zeiss, DP71; Olympus). Images were adjusted for level correction, brightness, and contrast with ImageJ.

### Phytohormone supplementation

To examine the effects of phytohormones on the reprogramming, bezsylaminopurine (BAP), naphthalenacetic acid (NAA), and synthetic strigolactone (GR24) were used. Observations were made using an inverted microscope (Axio Observer, Zeiss) equipped with a cooled CCD camera (Axio Cam 506 color, Zeiss).

### Statistical analysis

Regeneration rate was analyzed using exact tests of Fisher’s test and the Hardy-Weinberg equilibrium as implemented in the R package ‘HardyWeinberg’ version 1.5.5^30^. Positional effect along the proximal-distal axis for stem cell reprogramming of cell pairs was analyzed by binomial test. Differences in nuclear size were examined by Tukey-Kramer test.

## Electronic supplementary material

Supplementary Information

Supplementary Video 1

Supplementary Video 2

Supplementary Video 3

Supplementary Video 4

Supplementary Video 5

Supplementary Video 6

Supplementary Video 7
