# Supplementary material for: Cells reprogramming to stem cells inhibit the reprogramming of adjacent cells in the moss Physcomitrella patens
Source: Sci Rep. 2017 May 15;7:1909. doi: 10.1038/s41598-017-01786-1 (PMC5432521; doi:10.1038/s41598-017-01786-1)
Supplement: Supplementary file 1 — Supplementary Information [file 41598_2017_1786_MOESM1_ESM.pdf]

Cells reprogramming to stem cells inhibit the reprogramming of adjacent cells in the moss *Physcomitrella patens*

Yoshikatsu Sato, Nagisa Sugimoto, Tadayoshi Hirai, Akihiro Imai, Minoru Kubo, Yuji Hiwatashi, Tomoaki Nishiyama & Mitsuyasu Hasebe

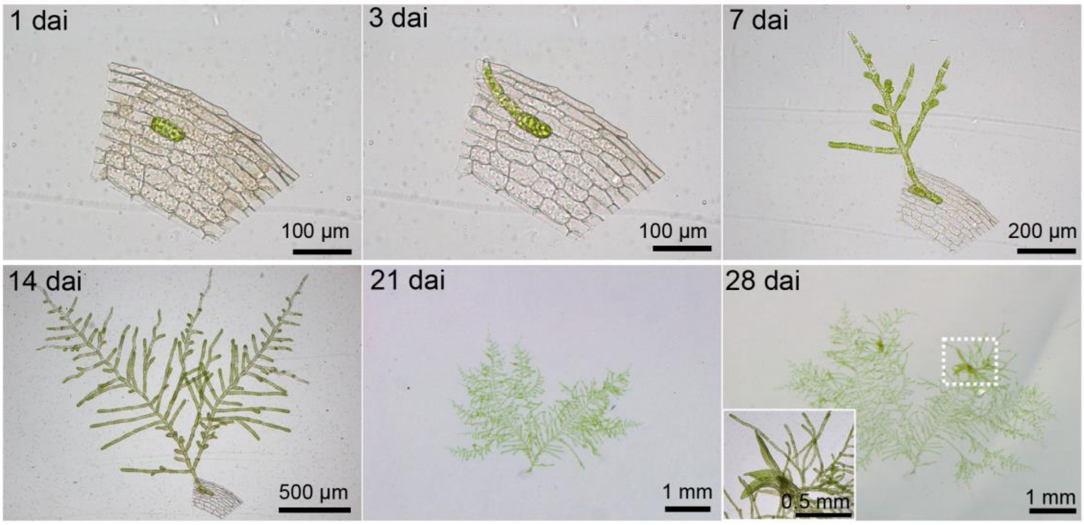

Supplementary Fig. 1: Regeneration of new gametophores from a single isolated leaf cell. Days after isolation and scales are indicated. Days after isolation (dai) were indicated in the upper left of each panel. Images were acquired using an inverted microscope Axiovert 200M (Zeiss) for 1-14 dai and inset of 28 dai and a stereomicroscope SZX16 (Olympus) for 21-28 dai. The inset on day 28 is flipped horizontally.

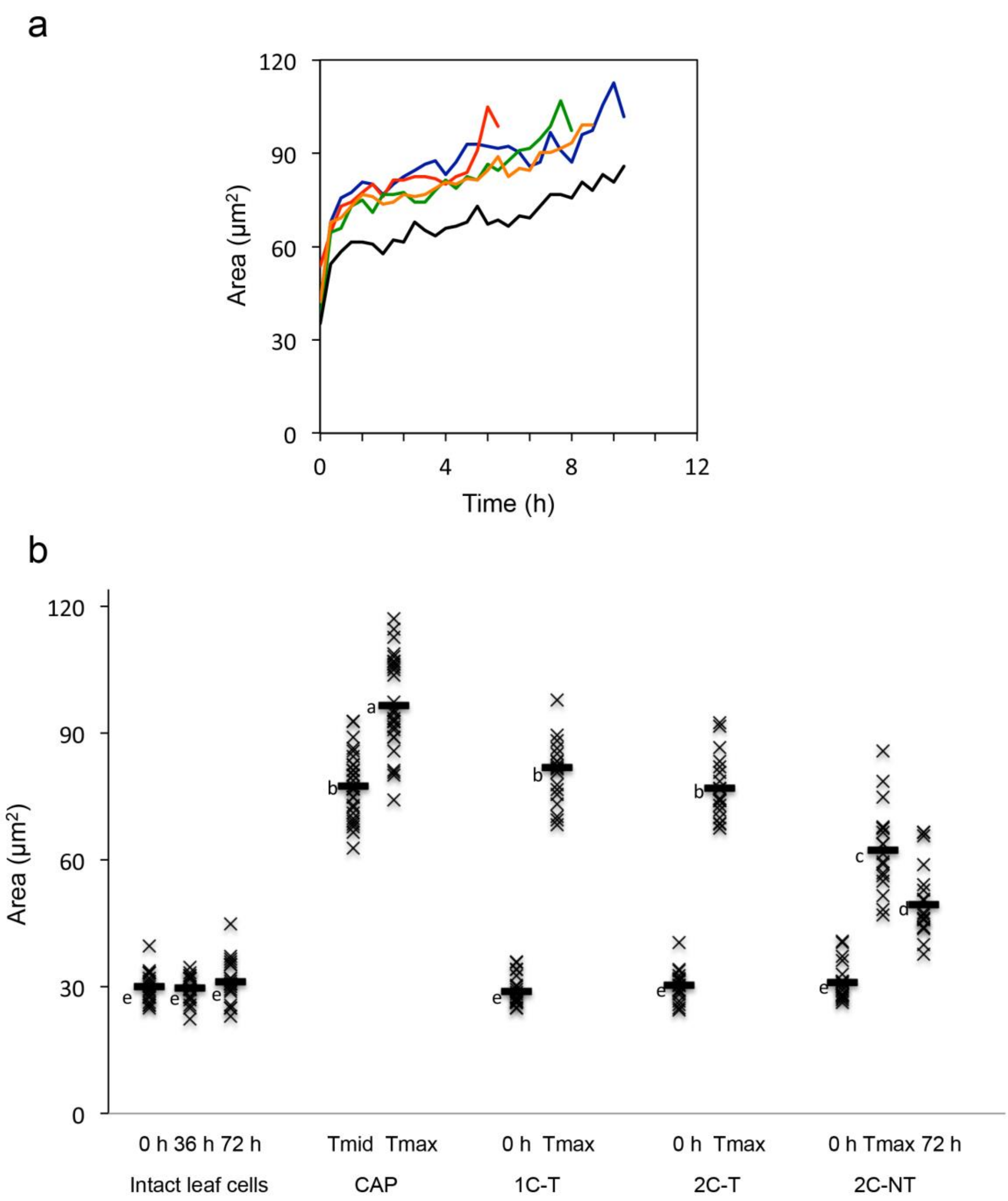

Supplementary Fig. 2: Changes of nuclear size in chloronema apical stem cells and isolated leaf cells. (**a**) Changes of nuclear size during the interphase of chloronema apical stem cells. Four representative chloronema apical stem cells are presented. Nuclear size immediately increases after cytokinesis (0 h) and continuously increase until following

cytokinesis (time at the end of each line). (**b**) Changes of nuclear size in intact leaf cells (n = 21), chloronema apical stem cells (CAP, n = 30), and isolated leaf cells. Tmid: the time at the middle point of interphase. Tmax: the time when nuclear size is maximized. Nuclear sizes were measured in cells with tip growth in single isolated cells (1C-T, n = 20) from 29 single isolated cells and leaf cells with tip growth (2C-T) or without tip growth (2C-NT) in isolated cell pairs with only one cell showing tip growth (n = 20) from 42 isolated pairs parallel to the leaf axis. Mean values are indicated by black bars. Characters (a-e) indicate significantly associated categories ($p < 0.01$ by the Tukey-Kramer test).

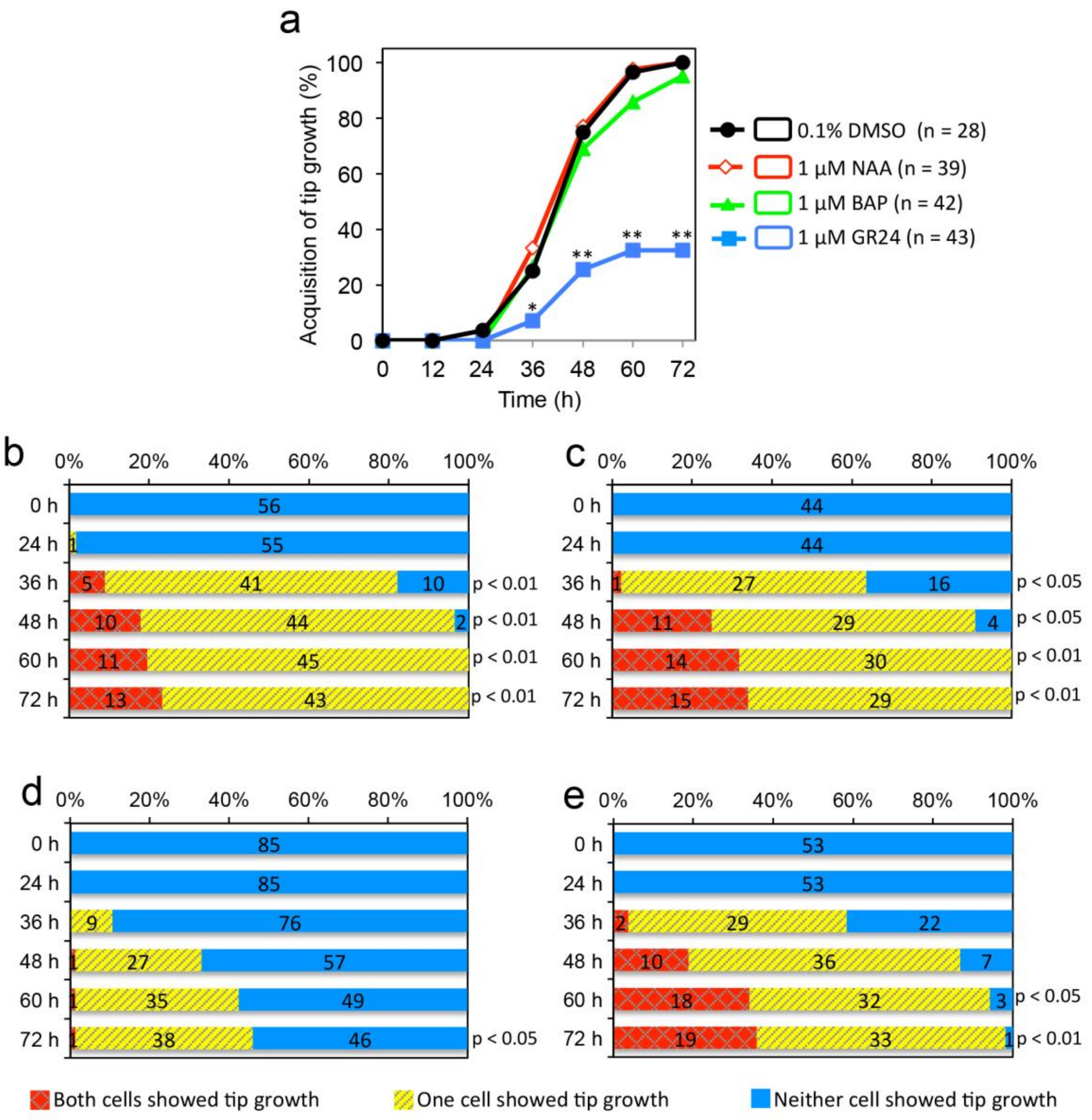

Supplementary Fig. 3: Effects of NAA, BAP and GR24 on cell reprogramming. (**a**) Percentages of cells with tip growth in single isolated cells. Statistical significance of difference from single isolated cells was examined by Fisher's test and p-values are shown as * ($p < 0.05$) and ** ($p < 0.01$). (**b-e**) Cell fates in isolated pairs of cells aligned parallel to the leaf proximal-distal axis in 1 µM NAA (**b**), 1 µM BAP (**c**), 1 µM GR24 (d), and 0.1 % DMSO (**e**) treated cells. Statistical significance was examined by Hardy-Weinberg equilibrium.

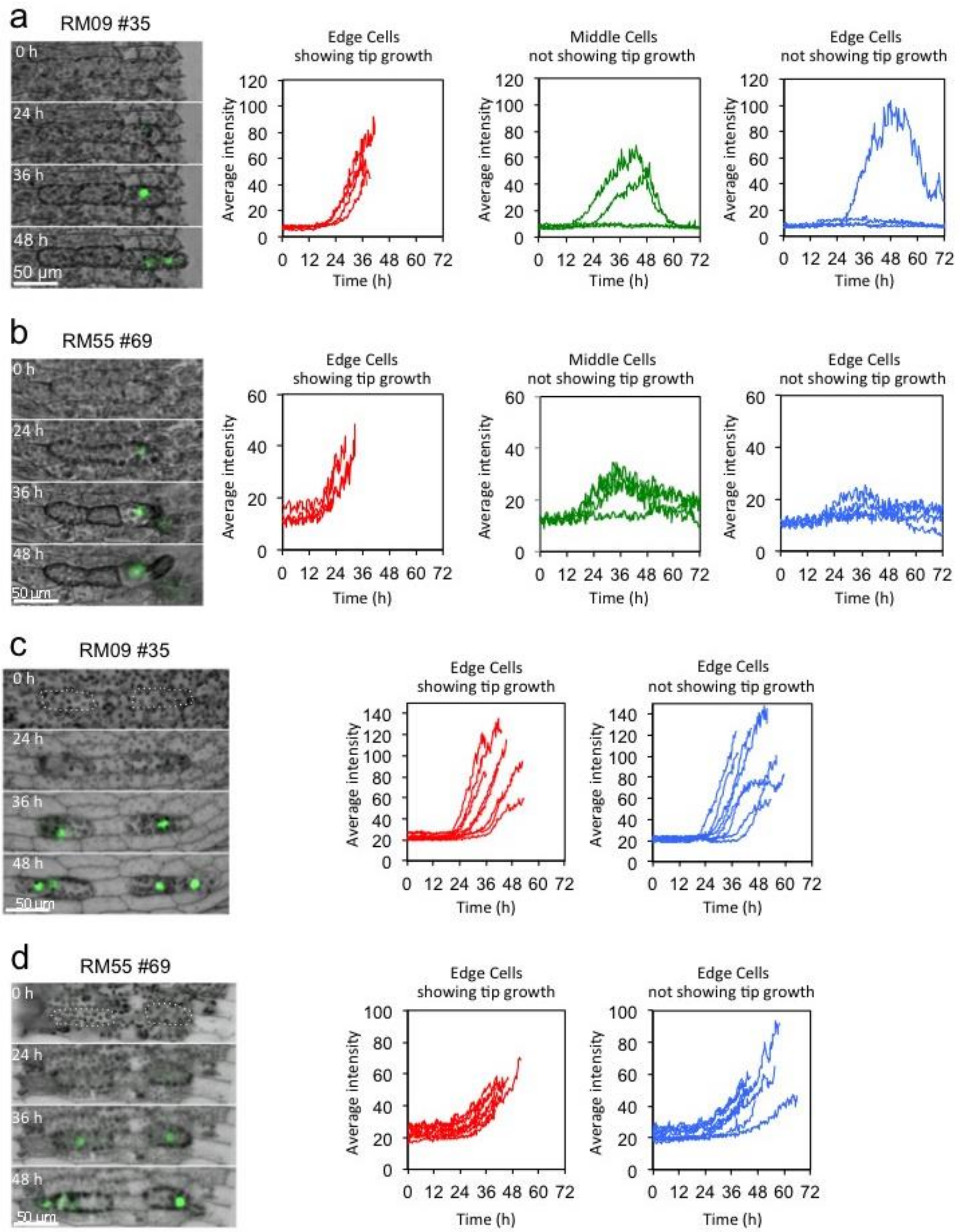

Supplementary Fig. 4: Acquisition of chloronema characters in isolated three aligned cells. (**a-d**) Representative images of RM09 #35 (**a, c**) and RM55 #69 (**b, d**) lines in one

cell showing tip growth when three cells were isolated with living middle cells (**a**, **b**) and in both cells showing tip growth when three aligned cells were isolated with dead middle cells (**c**, **d**). Twenty-one out of 34 and 13 out of 41 in triplet cells with living middle cells in RM09 and RM55 respectively showed tip growth only in an edge cell. Changes of GFP fluorescent signal are shown for four representative triplet cells until the time of tip growth or 72 h. Fifteen out of 18 and 13 out of 18 triplet cells with dead middle cells in RM09 and RM55 showed tip growth in both edge cells. Changes of GFP fluorescent signal are shown for eight representative triplet cells until tip growth starts. Edge cells in three aligned cells with dead cells were enclosed by dotted lines at 0 h. GFP (green) are overlaid with bright field images. Time after isolation and scales are indicated.

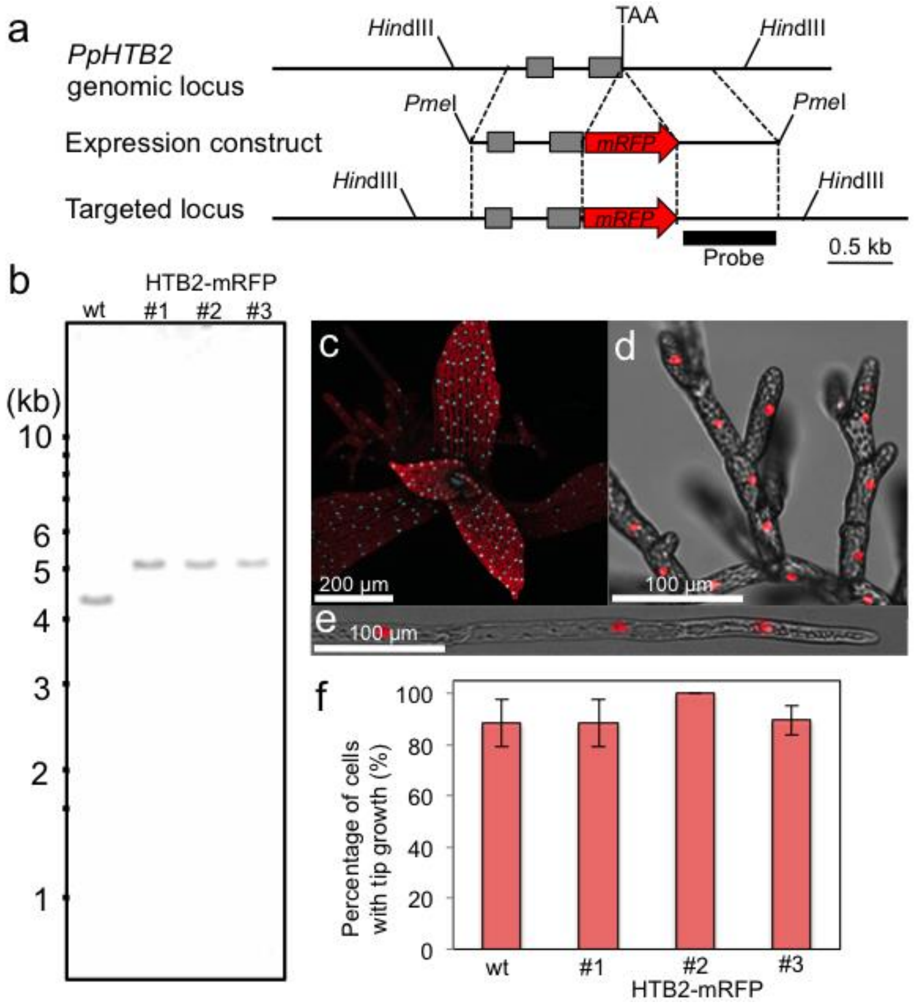

Supplementary Fig. 5: Construction of the HTB2-mRFP lines and expression of the HTB2-mRFP protein in protonema and gametophore cells. (**a**) Schematic diagram for the insertion of *mRFP* into the *histone H2B* (*HTB2*) locus. Red arrows denote *mRFP*. Boxes indicate putative translated exons of *HTB2*. A probe used in (**b**) is indicated. (**b**) DNA gel-blot analysis of targeted lines. Genomic DNA of the wild type (wt) and HTB2-mRFP #1, #2, and #3 lines were digested with *Hin*dIII. (**c-e**) mRFP fluorescence images in gametophore leaf cells (**c**), chloronema cells (**d**) and caulonema cells (**e**).

Autofluorescence of chlorophyll (red) and mRFP fluorescence (cyan) are overlaid in (**c**). mRFP fluorescence images (red) are overlaid with bright-field images in (**d**) and (**e**). (**f**) Percentage of leaves having at least one cell with tip growth in excised leaves (n = 20) at 48 h after excision. Error bars indicate SD from three biological replicates. Scales are indicated at the bottom left corner of each panel.

Supplementary Video 1. Formation of a putative chloronema apical cell from an isolated gametophore leaf cell. Time after isolation is indicated at the upper right corner of the movie. Selected frames (0, 48, and 60 h) are shown in Fig. 1a. Bar = 20 µm.

Supplementary Video 2. Promoter activity of protonema-specific gene *RM09* in a single isolated cell. Fluorescence images of GFP (green) are overlaid with bright-field images. Time after isolation is indicated at the upper left corner of the movie. Selected frames (0, 24, 36 and 48 h) are shown in Fig. 2a. Bar = 20 µm.

Supplementary Video 3. Promoter activity of protonema-specific gene *RM55* in a single isolated cell. Fluorescence images of GFP (green) are overlaid with bright-field images. Time after isolation is indicated at the upper left corner of the movie. Selected frames (0, 24, 36 and 48 h) are shown in Fig. 2b. Bar = 20 µm.

Supplementary Video 4. Effect of apical cell ablation on the remaining cell of a pair of isolated cells. The left cell was ablated 48 h after two-cell isolation. Time after isolation is indicated at the upper left corner of the movie. Selected frames (0, 24, 48, 72 and 96 h) are shown in Fig. 3j. Bar = 20 µm.

Supplementary Video 5. Changes in nucleus size during reprogramming in pairs of isolated cells in the HTB2-mRFP line. Fluorescence images of mRFP (red) are overlaid with bright-field images. Time after isolation is indicated at the upper left corner of the movie. Selected frames (0, 24, 48 and 72 h) are shown in Fig. 4b. Bar = 20 µm.

Supplementary Video 6. Promoter activity of protonema-specific gene *RM09* in pairs of isolated cells. Fluorescence images of GFP (green) are overlaid with bright-field images. Time after isolation is indicated at the upper left corner of the movie. Selected frames (0, 24, 36 and 48 h) are shown in Fig. 5a. Bar = 20 µm.

Supplementary Video 7. Promoter activity of protonema-specific gene *RM55* in pairs of isolated cells. Fluorescence images of GFP (green) are overlaid with bright-field images. Time after isolation is indicated at the upper left corner of the movie. Selected frames (0, 24, 36 and 48 h) are shown in Fig. 5b. Bar = 20 µm.
